# Associations of attitudes and social norms with experiences of intimate partner violence among married adolescents and their husbands in rural Niger: a dyadic cross-sectional study

**DOI:** 10.1186/s12905-022-01724-y

**Published:** 2022-05-18

**Authors:** Holly Baker Shakya, Beniamino Cislaghi, Paul Fleming, Ruti G. Levtov, Sabrina C. Boyce, Anita Raj, Jay G. Silverman

**Affiliations:** 1 Center On Gender Equity and Health, School of Medicine, University of California San Diego, 9100 Gilman Dr., San Diego, CA USA; 2 London School of Hygiene and Tropical Medicine Room, 330 LSHTM 15-17 Tavistock Place, London, WC1H 9SH UK; 3 Department of Health Behavior & Health Education, 3814 SPH I, 1415 Washington Heights, Ann Arbor, MI 48109-2029 USA; 4 Prevention Collaborative, Washington, DC USA

**Keywords:** Intimate partner violence, Social norms, Gender norms, Niger, Dyadic data, Couples data

## Abstract

**Background:**

Prior cross-sectional research suggests that both men’s and women’s attitudes towards intimate partner violence (IPV) are predictive of women’s IPV experience, although this can vary greatly by context. In general, women who have experienced IPV are likely to report attitudes accepting of it. Men who perpetrate IPV may also report attitudes accepting of it, although some research has found that there is not always an association. Studies that investigate these dynamics often conflate attitudes with social norms, or use attitudes as a proxy for social norms, given that valid measures on social norms are usually lacking. Here we conduct a secondary data analysis to ask how are men’s and women’s IPV-related attitudes associated with women’s reports of IPV and how are men’s and women’s perceived social norms associated with women’s reports of IPV.

**Methods:**

Dyadic data were collected from a representative sample of married adolescent girls and their husbands in 48 rural villages of the Dosso region of Niger (N = 1010). Assessments included logistic regression analyses of husbands’ and wives’ reports of individual attitudes towards IPV, and social norms based on husbands’ and wives’ perceptions of their communities’ beliefs related to gender roles and acceptability of IPV.

**Results:**

Eight percent of women in this sample reported IPV. We found that, consistent with other research, wives who have reported IPV are more likely to report attitudes in support of IPV, while for husbands whose wives report IPV, that relationship is insignificant. On the other hand, husbands who report that people in their community believe there are times when a woman deserves to be beaten are more likely to have perpetrated IPV, while for wives there is no association between the community norm and IPV reporting. Finally, wives who report that people in their community hold inequitable gender norms in general are more likely to have experienced IPV, while for husbands, community gender norms are not predictive of whether their wives have reported IPV.

**Conclusions:**

Our results are evidence that IPV prevention interventions focused solely on individual attitudes may be insufficient. Targeting and assessment of social norms are likely critical to advancing understanding and prevention of IPV.

## Background

The World Health Organization (WHO) estimates that about one of every three ever-partnered women has been a victim of sexual or physical violence by a male intimate partner, with the prevalence up to 70% in certain countries [1, 2]. Intimate partner violence (IPV) is not only a human rights violation, it is associated with multiple poor psychological and physical health outcomes for women, including death [3, 4]. Beyond the risks to the woman herself, children born to mothers who are victims of IPV are also at higher risk of negative health outcomes including malnutrition, respiratory and diarrheal illnesses, and neonatal and infant death [5–7].

While cultural and community level factors appear to support perpetration of IPV, not all men within such enabling environments perpetrate violence, suggesting that individual factors, such as exposure to family violence and individual attitudes, are also important determinants [1]. Prior cross-sectional research in multiple countries suggests that both men’s and women’s attitudes towards IPV are predictive of women’s reported IPV experiences, although this can vary greatly by context [8, 9]. In general, women who have experienced IPV are also more likely to report attitudes accepting of it [10–13]. In a large cross-sectional study looking at these associations within different countries, both men’s and women’s attitudes when included in the same models were independent predictors of women’s reported experience of IPV [8]. Consistent with these findings, most studies have found that men who perpetrate IPV tend to report attitudes accepting of IPV [8, 13, 14], although at least one study has failed to find such an association [15].

## Social norms and IPV

Though there are many studies of associations between men’s and women’s attitudes towards IPV and women’s experience of IPV, far fewer have clarified the role of social norms [16]. This is likely due, in part, to confusion over the many current definitions of social norms (vs. individual attitudes) and how they should be measured [17]. Linos and Kawachi [16] called for considering norms in IPV research, but the two papers they cite as examples [18, 19] measure social norms by aggregating individual attitudes.

To address this issue, current theoretical and empirical social psychology research has helped clarify the distinction between social norms and individual attitudes [20–22]. Rather than conceptualizing norms as a cluster of attitudes, social psychology defines norms as people’s beliefs about what individuals in a given group or society think to be appropriate and acceptable. We refer to these here as *second order beliefs*. In this stream of thought, norms are individuals’ beliefs about a) how others in their groups behave and b) of what behaviors those others approve [23, 24]. The distinction between attitudes and norms is important, as people might have a negative individual attitude towards a given behavior, and yet, with the goal of seeking others approval, engage in that behavior nonetheless. Following Cialdini, people’s beliefs about what others do are often referred to as *descriptive norms*, while people’s beliefs about what others approve and disapprove of are often called *injunctive norms* [25, 26].

Gender norms are distinct from social norms, and are an important aspect of the social factors sustaining IPV. Merging recent social norms theory with cognitive schema theory we follow Bem’s seminal work [27] and Strauss and Quinn [28] in understanding gender norms as shared cultural models that determine roles and appropriate behaviors for men and women within a specific setting. Social norms specific to particular behaviors are often sustained through culturally specific gender norms. Gender norms for women might intersect, particularly in highly patriarchal contexts, with local cultural models associating women with submissiveness, reliability, and servitude [29]. Bussey & Bandura [30] use a social cognitive model to outline three patterns through which gender norms can influence individuals’ behaviors: (a) social learning: observation and modeling of other’s behavior; this includes observing how others have been sanctioned for violations of expected behaviors; (b) being directly instructed by elders or those in authority regarding what is appropriate and expected; (c) direct social normative sanctioning, whereby personal deviations from the expectations are met with some form of social punishment.

## Research question

Given the relative dearth of literature on the effect of social norms on IPV, and the growing interest in using a social norms approach to address IPV, understanding the role that norms play in sustaining violence is crucial. This is of particular importance in a place like Niger, for which survey data are rare and analyses of factors that contribute to IPV in these contexts are lacking.

Niger—a land-locked country in Francophone West Africa—is the second most gender inequitable country in the world according to the UN’s Gender Inequality Index (ranked 154 out of 155 countries) [31, 32]. While comprehensive data on Niger is limited, the Demographic and Health Survey (DHS) provides a source of information on socio-demographics, health, fertility, and gender equity [33]. Gender inequity in Niger is reflected by differences in educational attainment: only 14% of women in Niger are considered literate, compared with 42% of men; and 64% of women have never been to school compared to 37% of men[33]. Niger has the highest rate of early marriage in the world, with the average age at first marriage for women being 15.7 years old [33]. The majority of girls (75%) are married by the age of 18 [34]. Not surprisingly, adolescent motherhood is very common: 40% of women have their first child between the ages of 15–18 [33]. Niger also has the world’s highest fertility rate, with an average of 7.6 births per woman of childbearing age[33]. Polygamy is common within Niger, and roughly one third (36%) of married women have co-wives[35].

This confluence of factors, which includes low levels of education, early marriage, and high fertility, makes it important to explore the vulnerabilities of young women in Niger, particularly their vulnerability to spousal violence. While 27% of men and 60% of women agree that there are times a wife deserves to be beaten [33], there are no official statistics on rates of IPV in Niger. Estimates of lifetime prevalence of physical or sexual IPV from neighboring countries range widely, from 12% in Burkina Faso and 16% in Nigeria, to 35% in Mali and 51% in Cameroon [36].

For this study, we conducted a secondary data analysis using dyadic survey data from adolescent wives and their husbands living in rural Niger, including measures of men’s and women’s attitudes and perceived social norms on violence against women; gender norms on appropriate roles for women; and women’s reports of IPV. Our analyses are constructed to help us understand how IPV-related attitudes, norms and behaviors intersect in a highly gender-inequitable setting. We ask how are men’s and women’s IPV related attitudes associated with women’s reports of IPV and how are men’s and women’s perceived social norms associated with women’s reports of IPV.

## Methods

### Data collection

Data were collected across 48 villages clustered within the Dosso, Doutchi, and Loga districts in the Dosso region of Niger as part of the baseline data collection for a cluster randomized control trial evaluating Reaching Married Adolescents, a contraception promotion and gender equality intervention led by Pathfinder International that targets married adolescent girls, their husbands, and communities. Details on data collection are published elsewhere [37, 38].

Twenty-five married female adolescents aged 13–19 years from each of the 48 villages (N = 1200) and their husbands (N = 1200) were selected. Eligibility criteria for the married female adolescents include: (a) ages 13–19 years old; (b) married; (c) fluent in Hausa or Zarma; (d) residing in the village where recruitment was taking place with no plans to move away or travel for more than 6 months during the next 18 months; (e) not currently sterilized; and (f) providing informed consent to participate.

The instrument is available online [38].

### Measures

Survey items for wives and husbands were close-ended questions constructed to reflect the experiences, meanings, and language of the target population related to gender, violence, and reproductive health. Questions were based on formative research findings, prior work of the project team, and existing validated instruments for men and women in low resource settings, including the DHS [33]. The surveys were developed in English by UCSD, translated into French, back-translated to English for content reliability check, programmed in French, and verbally administered in Hausa or Zarma. As Hausa and Zarma are rarely expressed in written form, this is the translation protocol that has been most commonly utilized in the Niger context [33, 39]. To ensure consistency, research assistants were trained on each item with an intended, agreed upon translation.

Dependent/Outcome Variable: Lifetime IPV Perpetrated by Husband (yes or no).

We used questions from the DHS domestic violence module to assess married adolescent girls’ experiences of IPV [40]. Girls were asked to report via 6 survey items whether, in the history of her marriage, her husband had ever pushed her, shaken her or thrown something at her; slapped her; twisted her arm or pulled her hair; hit her with his fist or something that could hurt her; kicked her, dragged her, or beat her up; or choked her or tried to burn her. We dichotomously coded physical IPV perpetration, with “yes” indicating an affirmative response to any of these questions. Protocols modeled after the World Health Organization’s guidelines for conducting research on violence against women [41] were implemented to protect the safety and confidentiality of women participating in the study.

Independent variables: Attitudinal and social normative predictors.

Individual IPV acceptance (yes or no).

We adapted questions from the DHS men’s and women’s questionnaires to assess individual attitudes expressing acceptance towards IPV [42]. Husbands and wives were both asked to report, “In your opinion, is a husband justified in hitting or beating his wife in the following situations: (a) If she goes out without telling him? (b) Uses a family planning method without telling him? (c) Argues with him? (d) Refuses to have sex with him? (e) Burns his food?” Answer choices were either “yes” or “no”. Consistent with previous research, we coded a person as positive on IPV acceptance if they answered positively to any of the five questions [8, 43]. Alpha on the full measure for wives was 0.82 and 0.77 for husbands.

Social norms: gender role second order beliefs (GRSB):

Both husbands and wives were asked a series of questions to understand second order social beliefs regarding the roles of men and women in their communities, with a focus on traditionally patriarchal, gender inequitable expectations for men’s and women’s behavior. The items were adapted from the Gender Equitable Men (GEM) scale [44, 45] to better reflect second order beliefs (for specific questions please see the online appendix). The highest score possible was seven, with a higher score reflecting perceptions of more inequitable community beliefs. Cronbach’s alpha for the scale was 0.79 for husbands and 0.88 for wives.

Social norms: violence against women second order social beliefs (VAWSB):

The GRSB included a question on the community-level acceptability of violence against women: people in this village believe that there are times when a woman deserves to be beaten. Because the outcome of interest was IPV, we removed the violence question from the main GRSB, and included it as a separate predictor in our models. Because the VAWSB second order social beliefs question is not a scale, we retained it as a three-factor categorical question, including one category for those who replied “Don’t Know.”

### Statistical methods

*Models include the following sociodemographic controls*: husbands and wives ages, levels of education, having received a Quranic education, wives age at marriage, family wealth, food insecurity, number of children born to the couple, whether or not they live in an extended family, number of wives the husband has, wife’s engagement in agricultural work, language and district. For more detail on these measures please see the online appendix.

We used logistic regression on dyadic observations including both husbands’ and wives’ measures to assess the odds of a wife reporting having ever experienced IPV given the attitudinal and normative predictors plus sociodemographic covariates. We first ran bivariate models assessing the association of the attitudinal and normative predictors with wives’ IPV reporting then ran multivariate models including any attitudinal and normative predictors with a p-value of < 0.10 plus all sociodemographic covariates. Our initial models were separate for husbands and wives, with the final model combining husband’s and wife’s predictors below the cut off (p < 0.10) in order to see whether, consistent with previous research [46], both husbands’ and wives’ measures are independently associated with the outcome after controlling for the other. This unique dyadic dataset allowed us to examine how factors related to both the husband’s and wife’s attitudes and perceptions of community behaviors are associated with IPV reported by women.

## Results

Table 1 shows the summary statistics for our data. After removing data with missing values and non-matched wife-husband dyads, our final sample size was 1010 husband-wife dyads. The average age of the wives in our data was just over 17 years old, with the average age at marriage for wives just over 14 years. The average husband’s age was slightly less than 26 years. In 41% of the couples, the wife reported engaging in agricultural work.

**Table 1:** Summary statistics of couple level dyadic data, Niger N = 1010

	Mean	SD	%
Wife’s age (13–19)	17.31	1.53	
Husband’s age (15–53)	25.58	5.36	
Wife’s education (0–3)	0.50	0.79	
Husband’s education (0–3)	0.73	0.89	
Wife’s Quranic schooling (Yes vs no)			26%
Husband’s Quranic schooling (Yes vs no)			34%
Wife’s age at marriage (10–19)	14.20	1.84	
Household assets (0–6)	2.07	1.17	
Food insecurity			20%
Wife’s agricultural labor			42%
Number of children (0–5)	0.93	0.96	
Live with extended family			81%
Husband’s number of wives (0–4)	1.15	0.40	
Tribe Hausa			31%
Tribe Zarma			69%
Tribe Tuareg			0.05%
District Dosso			32%
District Doutchi			33%
District Loga			35%
Wife reports of IPV			8%
Wife’s IPV acceptance (binary)			66%
Husband’s IPV acceptance (binary)			51%
Wife’s gender role second order social beliefs scale (0–7)	5.99	1.66	
Husband’s gender role second order social beliefs scale (0–7)	5.84	1.62	
Wife VAWSB second order social beliefs—yes			54%
Wife VAWSB second order social beliefs—doesn’t know			1%
Husband VAWSB second order social beliefs—yes			58%
Husband VAWSB second order social beliefs—doesn’t know			8%

Approximately 8% of wives reported ever having experienced IPV at the hands of their husbands, with the most prevalent behavior being slapping (6% of respondents) and the least prevalent behaviors being choking and kicking (1% of respondents for each). The majority of respondents, both husbands and wives, reported acceptance of IPV, with wives being more likely to report acceptance of it in at least one situation compared to husbands (66% vs 51%, p < 0.01). On average women were likely to endorse 1.9 items on the individual IPV attitude scale, while men were likely to endorse 1.3. The average score on the GRSB was slightly higher for wives (5.99) than for husbands (5.84) (p = 0.04), indicating that, across gender, both men and women had strong perceptions that their community supported highly gender segregated roles for men and women, consistent with traditional patriarchal viewpoints. For the VAWSB, 58% of husbands reported that people in their community believe that there are times when women deserve to be beaten, which was slightly higher than the 54% of wives who reported this same norm (p < 0.01). Also of note is that 8% of husbands replied that they did not know the answer to that question, while only 1% of wives reported this. While wives’ and husbands’ reports of these attitudes and social beliefs were overall similar across the total population, concordance within couples was not particularly high. The within couple correlation for the GRSB was only 8%, 12% for attitudes accepting of IPV, and 25% for second order beliefs regarding the use of violence against women. Using the criterion proposed by Landis and Koch, [47, 48] the level of correlation within couples on these measures varied from poor to barely fair (Poor < 0.01, Slight 0.01–0.20, Fair 0.21–0.40, Moderate 0.41–0.60, Substantial 0.61–0.80, Almost Perfect 0.81–1.00).

Table 2 includes results of a multivariate logistic regression model of the social and demographic predictors of IPV in the sample population. We found that wives who engaged in agricultural work outside of the home, had a higher level of education, and lived in the Loga district were less likely to experience IPV when controlling for other variables in the model, but that a husbands’ individual socio-demographic characteristics were not significantly associated with wives’ reports of IPV.

**Table 2:** Socioeconomic and demographic predictors of wives’ reports of ever having experienced IPV, Niger, N = 1010

	Beta	SE	P
Wife’s age (13–19)	− 0.01	0.11	0.916
Husband’s age (15–53)	0.00	0.03	0.977
Wife’s education (0–3)	− 0.34	0.19	0.072
Husband’s education (0–3)	0.03	0.15	0.832
Wife’s Quranic schooling (yes vs no)	− 0.29	0.31	0.351
Husband’s Quranic schooling (yes vs no)	0.38	0.27	0.160
Wife’s age at marriage (10–19)	− 0.03	0.09	0.760
Household assets (0–6)	− 0.1	0.11	0.371
Food insecurity (yes vs no)	0.00	0.28	0.997
Wife agricultural labor (yes vs no)	− 0.93	0.30	0.002
Number of children (0–5)	0.20	0.17	0.244
Live with extended family (yes vs no)	− 0.47	0.38	0.218
Husband’s number of wives (0–4)	− 0.57	0.47	0.222
Tribe Zarma (Ref: Hausa)	1.14	0.64	0.076
Tribe Tuareg (Ref: Hausa)	1.3	1.23	0.289
District Doutchi (Ref: Dosso)	1.02	0.65	0.116
District Loga (Ref: Dosso)	− 0.66	0.33	0.044

In Table 3 we begin to consider the attitudinal and social normative predictors of wives’ reports of IPV victimization through bivariate analyses. We found that husbands’ acceptance of IPV was not associated with wives’ IPV reports, while wives’ acceptance of IPV was highly correlated with their reports of IPV. The odds that a woman reported IPV increased 3.22 (95% CI 1.72–6.03) times if she herself reported acceptance of IPV in one or more of the contexts asked in the survey. In contrast to the attitudinal results, both the wife’s and the husband’s GRSB measures were significantly associated with IPV. The VAWSB measure, however, was significantly associated with IPV for husbands but not for wives. When husbands agreed that people in their community believe that there are times when women deserve to be beaten, the odds that his wife reported ever having experienced IPV were 2.10 times higher (95% CI 1.19–3.70) than when husbands did not agree. Interestingly, those who replied “I don’t know” were also more likely to have perpetrated IPV according to their wife’s reports than those who replied that they did not agree.

**Table 3:** Attitudinal and normative associations with IPV, bivariate associations^1^

	Model 1 husbands			Model 2 wives		
	Beta	SE	P	Beta	SE	P
IPV acceptance (binary)	0.31	0.23	0.176	**1.17**	**0.32**	** < 0.000**
VAWSB social beliefs—no						
VAWSB social beliefs—yes	**0.74**	**0.29**	**0.009**	0.26	0.25	0.263
VAWSB social beliefs—doesn’t know	**0.84**	**0.45**	**0.061**	NA^2^	NA	NA
GRSB scale (0–7)	**0.15**	**0.08**	**0.083**	**0.43**	**0.13**	**0.001**

Bolded items fall below the cut-off of *p* < 0.10 and are carried forward into the next model

^1^Attitudinal and normative predictors below the cut off of p < 0.10 are included in the multivariate analysis

^2^Cell size too small for analysis

Table 4 shows the multivariate models in which we brought forward the attitudinal and social normative predictors that were at the cutoff p value of p < 0.10 in the bivariate models plus all sociodemographic covariates. Model 1 shows the results based on husbands’ reports. In the multivariate models the VAWSB measure retained significance, however the gender role second order social beliefs scale lost significance. When husbands agreed that people in their community believed that there were times when a woman deserves to be beaten, their wives had 2.08 times the odds (95% CI 1.13–3.81) of reporting spousal IPV than when husbands disagreed. Model 2 shows the results of the wives’ analyses. Wives who reported individual attitudes accepting of IPV had 2.97 times (95% CI 1.56–5.68) the odds of experiencing IPV than those who reported individual attitudes not accepting of it. We found that the women’s score on the GRSB was also predictive. For each standard deviation increase in the woman’s score on the GRSB, the odds that she reported IPV increased by 2.14 times (95% CI 1.60–2.87). We also see in both of these models that wife’s education is still negatively associated with IPV reporting although it is only marginally significant (p = 0.06 and p = 0.08). Wife’s agricultural work, however, remains strongly significant in both models, suggesting a strong negative association with women’s agricultural labor and IPV in these contexts.

**Table 4:** Second order beliefs associations with ever having experienced IPV^1,2^

	Model 1 Husbands			Model 2 Wives		
	Beta	SE	P	Beta	SE	P
**IPV acceptance (yes vs no)**				**1.09**	**0.33**	**0.001**
VAWSB social beliefs (ref: no)						
**VAWSB social beliefs—yes**	**0.73**	**0.31**	**0.018**			
VAWSB social beliefs—doesn’t know	0.71	0.47	0.129			
GRSB ^*^	0.06	0.10	0.580	**0.47**	**0.15**	**0.002**
Wife’s age (13–19)	− 0.05	0.11	0.674	0.01	0.11	0.920
Husband’s age (15–53)	0.01	0.03	0.825	0.00	0.03	0.954
Wife’s education (0–3)	− 0.35	0.19	0.070	− 0.33	0.19	0.080
Husband’s education (0–3)	0.01	0.15	0.971	0.02	0.16	0.910
Wife’s Quranic schooling (yes vs no)	− 0.27	0.31	0.382	− 0.33	0.32	0.311
Husband’s Quranic schooling (yes vs no)	0.32	0.27	0.236	0.35	0.28	0.206
Wife’s age at marriage (10–19)	− 0.02	0.09	0.861	− 0.07	0.09	0.448
Household assets (0–6)	− 0.12	0.11	0.277	− 0.19	0.11	0.091
Food insecurity (yes vs no)	0.00	0.29	1.000	0.13	0.30	0.649
Wife’s agricultural labor (yes vs no)	− 0.91	0.31	0.003	− 0.78	0.31	0.011
Number of children (0–5)	0.24	0.18	0.167	0.14	0.18	0.448
Live with extended family (yes vs no)	− 0.47	0.38	0.216	− 0.44	0.38	0.251
Husband’s number of wives (0–4)	− 0.57	0.47	0.230	− 0.59	0.47	0.220
Tribe Zarma (Ref: Hausa)	1.31	0.66	0.050	0.95	0.64	0.141
Tribe Tuareg (Ref: Hausa)	1.16	1.24	0.351	0.95	1.21	0.436
District Doutchi (Ref: Dosso)	1.18	0.67	0.076	0.90	0.65	0.165
District Loga (Ref: Dosso)	− 0.63	0.33	0.059	− 0.72	0.33	0.031

Bolded items fall below the cut-off of *p* < 0.10 and are carried forward into the next model

^1^Attitudinal and normative items included were below the p-value cutoff of 0.10 in the bivariate analyses (shown in Table 3)

^2^All socioeconomic and demographic variables retained in models as controls regardless of previous p-value

**Fig. 1:**
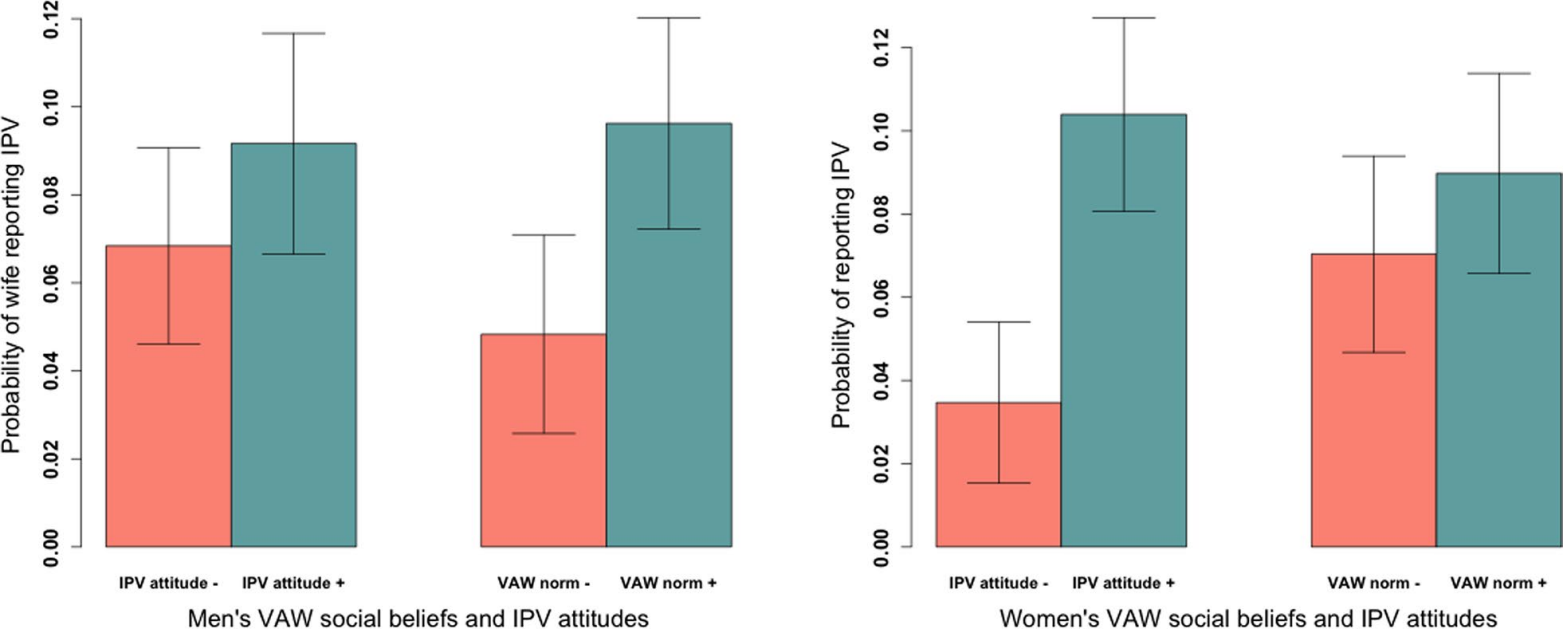
The probability of wife’s reported IPV by gender, IPV acceptance attitudes, and VAWSB

In Table 5 we combined the husbands’ and wives’ final models to assess whether the husbands’ and wives’ measures were independently associated with reported IPV after accounting for the attitudes and the social beliefs of the other. Finally, to ensure that the scales for IPV acceptance and GSR were comparable, we re-ran all of our models using the IPV beliefs scale as a continuous measure rather than as a binary measure (not shown). The results did not change, however we found that the probability of a wife reporting IPV increased with each point increase in her IPV attitudes scale. The association of IPV acceptance and IPV reporting was not only binomial but continuous. Figure 1 shows the probability of wife’s reported IPV by gender, IPV acceptance attitudes, and VAWSB.

**Table 5:** Combined model of men’s and women’s significant attitudinal and normative predictors of women’s reported IPV

	Combined model		
	Beta	SE	P-value
**Wife’s IPV acceptance (binary)**	**1.13**	**0.34**	**0.001**
**Husband’s VAWSB second order social beliefs (Ref: No)**			
**VAWSB second order social beliefs—yes**	**0.73**	**0.31**	**0.017**
VAWSB second order social beliefs—doesn’t know	0.64	0.48	0.181
**Wife’s GRSB scale (0–7)**	**0.42**	**0.15**	**0.017**
Wife’s age (13–19)	− 0.02	0.11	0.883
Husband’s age (15–53)	0.01	0.03	0.771
Wife’s education (0–3)	− 0.33	0.19	0.083
Husband’s education (0–3)	− 0.01	0.16	0.942
Wife’s Quranic schooling (yes vs no)	− 0.30	0.32	0.348
Husband’s Quranic schooling (yes vs no)	0.30	0.28	0.281
Wife’s age at marriage (10–19)	− 0.07	0.09	0.470
Household assets (0–6)	− 0.22	0.12	0.058
Food insecurity (yes vs no)	0.14	0.30	0.635
Wife’s agricultural labor (yes vs no)	− 0.80	0.31	0.010
Number of children (0–5)	0.17	0.18	0.332
Live with extended family (yes vs no)	− 0.49	0.39	0.210
Husband number of wives (0–4)	− 0.61	0.48	0.210
Tribe Zarma (Ref: Hausa)	1.02	0.67	0.130
Tribe Tuareg (Ref: Hausa)	0.74	1.22	0.545
District Doutchi (Ref: Dosso)	0.96	0.67	0.153
District Loga (Ref: Dosso)	− 0.70	0.34	0.037

Bolded items fall below the cut-off of *p* < 0.10 and are carried forward into the next model

## Discussion

The current study utilized dyadic data from married adolescent wives and their husbands living in rural Niger to look at the attitudinal and social normative predictors of IPV. This is, to our knowledge, one of the first studies to consider social norms as a predictor of IPV beyond the more commonly measured individual attitudes adopted as a proxy for norms. Our findings suggest that social norms regarding IPV may be important determinants of IPV perpetration, independent of individual attitudes, indicating that measures focused solely on individual attitudes may be insufficient to capture higher order social determinants of IPV within communities.

Our study offers important insights into the social context of IPV in Niger, as well as a glimpse into the gender inequalities that may support it. Consistent with Niger’s ranking as a highly gender inequitable country, participants in this survey rated their communities as highly gender inequitable, with strongly segregated gender roles. Because the GRSB scale used in this study has not been utilized in other settings, however, it is not possible to compare the means here to what would be found elsewhere. Nevertheless, the mean GRSB scores for both husbands and wives were extremely high, the mean age of marriage for wives was 14, and the average age of the husband was close to 9 years older than his wife; all well-established markers of gender inequity.

Our results showed that wives who report ever having experienced IPV in their marriage were significantly more likely to report acceptance of IPV versus wives who did not report having experienced IPV. We also found that wives who believe that their communities are gender inequitable and supportive of strongly segregated roles for men and women were more likely to have experienced IPV. Because the data is cross-sectional we do not know the directionality of these associations, but it is important to consider possible dynamics by which they occur. Existing evidence suggests that girls and women who are exposed to family violence as children are more likely to be victims of IPV as adults [49, 50]. In other words, women who have grown up experiencing or witnessing violence may see it as normal, so when violence occurs within their own relationships, they might be more likely to express attitudes accepting of it. Recent longitudinal work in India suggests that this may not be the case, at least for women who experience IPV in the short-term; women’s change in IPV attitudes over time did not change the likelihood that they reported IPV, while acceptance of IPV among women decreased significantly after experiencing IPV for the first time in this Indian context [13]. Similarly, we cannot determine with certainty if wives who experience IPV live in less equitable environments, or if they perceive their environments as being less equitable because they have suffered from violence. It is possible that both dynamics are occurring simultaneously, and longitudinal research would help further clarify our findings.

Even though we measured the association of gender inequitable norms with IPV experience, we do not know the extent to which the existence of these norms might be directly supporting the violent behavior. Furthermore, the question we used to understand norms regarding violence against women does not directly measure the existence of a norm that supports wife beating. The question we used asked respondents to agree or disagree with the statement “People in my village think that there are times when a woman deserves to be beaten”; in other words, participants believe that people in the community believe that women should be beaten for behavior that is considered unacceptable. The specific behaviors that warrant the sanction of beating are not specified- only that there are some circumstances in which a woman’s behavior should be sanctioned with beating. If a woman deserves to be beaten as a result of violating a norm, there will have to be someone who is responsible for that sanctioning. In most patriarchal contexts, that person would be the father, or in the case of married women, the husband, on whom the woman’s sanctionable behavior would, from the perspective of the community, directly reflect [51]. Husbands who believe that others in their community think that there are times when women should be beaten are the most likely to have beaten their wives regardless of their own beliefs regarding IPV.

Does believing that others expect someone to beat norm-violating wives encourage husbands to perpetrate IPV, or are husbands who perpetrate IPV more likely to register positive approval for their behavior, or is it some combination of the two? Again, with cross-sectional data we do not know the answer to that question, although these results may provide some clues to the poor correlation in the wives’ response regarding the social environment of the community compared to that of the husbands. If husbands believe that there are contexts in which a woman’s norm violation is enforceable with violence, and if that expectation is associated with his perpetration, then it seems likely that wives who are perceiving an inequitable environment might be tapping into a legitimate aspect of their communities that is contributing to violence against women within these communities. The GRSB scale measures expectations around multiple normatively reinforced behaviors specific to women and their roles within their families. Wives who have reported IPV may be reflecting on their own experiences with the use of violence to reinforce these expectations. While husbands’ perception of their social environment is not predicting their IPV behavior, wives who are experiencing that behavior from their husbands are perceiving that their environment sanctions this violence. For husbands, the salient factor seems to be the more specific, individual expectation regarding violence against women, rather than the overall environment of inequity. While future research will help us understand these dynamics more completely, our results nevertheless provide evidence that perceived community judgement regarding IPV is strongly associated with its occurrence and that effective interventions to reduce IPV in these communities very likely need to address the broader social context of this behavior.

Also interesting to note is that more husbands than wives answered “don’t know” to the questions regarding social beliefs, potentially contributing to the poor correlation in scores between couples. Husbands who answered “Don’t know” to the VAWSB social beliefs question were also likely to have perpetrated IPV, although the association lost significance in the multivariate models. Husbands were more likely than wives to report “don’t Know” to many of the social beliefs questions. Do these men not know what is expected in their communities regarding these sorts of behaviors? Or are these “don’t know” responses a form of response bias, as men implicitly understand that negative answers would be an outright lie, while positive answers would be revealing deep inequity within their communities? Noting changes to “don’t know” responses as a results of intervention efforts will be an important future avenue for this sort of norms research.

The methods and analyses currently presented may inform the development of social norms strategies. Norms research using quantitative tools is still a developing field [17]. The measures used in this study can be improved upon by using items that more explicitly tap into specific constructs from the social norms theoretical framework, and by testing them in different settings. Studies such as ours can also be strengthened through the use of social network data collection and analytic techniques, that provide far more resolution in the identification of the social reference groups that are salient for specific behaviors [43, 52]. Cultural models can be understood through the use of techniques such as cognitive-affective mapping [53] and cultural domain analysis [54], which can provide the foundation for understanding the interdependent gender norms. The measures used here, while not specifically uncovering cultural models, provide important information regarding the social contexts of IPV within these rural communities in Niger.

### Limitations

Our analysis has limitations. First and most obvious, perhaps, is the fact that the data are cross-sectional, so longitudinal associations and assessment of temporality are not possible. Second, as in almost all IPV research, IPV experience is a self-reported measure provided by wives who may be biased in their responses. The reported occurrence of lifetime IPV at 8% is low compared to similar contexts, and may be reflective of response bias. However, it is also necessary to remember that our respondents are adolescent girls, and so the extent of their possible exposure to IPV, simply based on age, is limited. Third, the second order social beliefs measures themselves are somewhat opaque in terms of tapping into specific constructs from social norms theory, which slightly complicates the interpretations of the findings. Future research can build off this scale to more theoretically reflect the specific constructs of social norms theory. Finally, our findings only reflect one specific region of Niger, Dosso, and so are not necessarily representative of the entire country.

## Conclusion

Despite these limitations, this study is unique in the use of dyadic data from a highly gender inequitable context, from which quantitative research like this is extremely rare, and in the use of both attitudinal and gender normative measures specific to the social context regarding IPV. Analyses from understudied populations like that of Niger are crucial for developing a more diverse understanding of factors that are associated with IPV, and how they may be the same or differ across contexts. Interest in social norms research has increased rapidly over the past several years, as health and development researchers realize that individual-based approaches may be insufficient in contexts in which behaviors are determined by broader social forces. Our study offers valuable evidence that perceptions of community expectations regarding IPV are related to this violence as a behavior, indicating that interventions that attempt to address IPV may be unlikely to succeed without addressing broader community-level social expectations. Longitudinal research and research that includes more nuanced measures to capture norms related to IPV will be crucial to furthering our understanding of these important dynamics.

## Data Availability

Data and materials are available upon request. Please send inquiries to the Corresponding Author.

## Abbreviations

WHO: World Health Organization
IPV: Intimate partner violence
DHS: Demographic and health survey
UN: United Nations
GRSB: Gender role second order beliefs
VAWSB: Violence against women second order beliefs
GEM: Gender equitable men
IRB: Institutional Review Board
